# Modeling of Vector-Borne Disease Across Governorates and Districts in Oman, 2020–2024

**DOI:** 10.3390/diseases14060196

**Published:** 2026-05-31

**Authors:** Abdullah Al-Manji, Adil Al Wahaibi, Amal Al Malehi, Mohammed Al-Azri, Moon Fai Chan

**Affiliations:** 1 Department of Family Medicine and Public Health, College of Medicine and Health Sciences, Sultan Qaboos University, Muscat 123, Oman; s132154@student.squ.edu.om (A.A.-M.); a.malehi@squ.edu.om (A.A.M.); mhalazri@squ.edu.om (M.A.-A.); 2 Ministry of Health, Muscat 100, Oman; adilwahaibi@gmail.com

**Keywords:** dengue, vector-borne disease, PLS-SEM, multigroup analysis

## Abstract

Introduction: Oman has transitioned from travel-related dengue cases to local outbreaks since 2018, with heterogeneous patterns across governorates and districts. Understanding how climate, population, and vector indicators jointly shape dengue risk at different administrative levels is essential for targeted control. Methods: This study compiled weekly data (2020–2024) on dengue cases, mosquito surveillance, climate, and population from national sources. Using Partial Least Squares Structural Equation Modelling (PLS-SEM) in SmartPLS v4, we modelled constructs for Weather, Population, Vector, and Vector-borne Disease (VBD). Measurement quality was assessed using various statistics and with 5000-sample bootstrapping. Multigroup Analysis (MGA) with permutation and Measurement Invariance of Composite Models (MICOM) tested invariance and compared structural paths across governorates (Muscat, North Al Batinah, Ad Dakhiliyah) and districts (Seeb, Sohar, Bahla). Results: Vector abundance mediated climate and population effects on dengue, with marked spatial heterogeneity. At the governorate level, the Vector → VBD path was strongest in Ad Dakhiliyah (β ≈ 0.436) and negligible in Muscat (β ≈ −0.021); indirect effects from Population and Weather to VBD were significantly higher in Ad Dakhiliyah than comparators. At the district level, Bahla showed stronger Vector → VBD and Weather → Vector relationships than Seeb and Sohar, while Seeb exhibited low explanatory power across paths. MICOM indicated partial measurement invariance, suggesting caution in cross-group comparisons. Conclusions: Dengue risk in Oman is primarily vector-driven but differs by setting. Inland/rural areas are more sensitive to climate–vector dynamics, requiring enhanced surveillance and climate-informed early warning. Urban centers may need models incorporating mobility and behavior. Findings support localized interventions and the integration of trap positivity and density into district-level prediction and control.

## 1. Introduction

Vector-borne diseases (VBDs), particularly dengue fever, are an escalating public health concern in Oman and the broader Middle East and North Africa (MENA) region [1,2]. While dengue in Oman was historically linked to travel-related cases, this changed in 2018, when the first locally transmitted infections were reported in the Seeb district of Muscat Governorate [3]. Since then, recurrent outbreaks in 2022 have underscored the growing potential for sustained endemic transmission, driven by the presence of the *Aedes aegypti* mosquito [4,5]. This epidemiological transition mirrors global trends, where complex interactions among climate variability, urbanization, human mobility, and socio-environmental factors increasingly shape dengue transmission [6,7,8]. In the MENA region specifically, a recent systematic review [9] confirms that overlapping demographic and ecological risk factors, including rapid urban expansion, poor sanitation, and insufficient vector control measures, influence mosquito-borne disease (MBD) dynamics [10,11]. In Oman, these vulnerabilities are particularly pronounced in rapidly urbanizing and densely populated areas such as Seeb. A recent study conducted by the research team in Seeb found that population-related factors, including population density and national composition, had a greater total effect on dengue incidence than climate or vector-related variables alone [12]. Complementary work employing hierarchical Bayesian models further demonstrated the value of incorporating lagged climate and entomological variables in forecasting dengue trends, highlighting the utility of temporally structured models for early warning systems [13]. Although previous epidemiological, regression-based, and forecasting studies have improved the understanding of dengue transmission in Oman and the wider MENA region, several important limitations remain. Many existing approaches primarily focus on direct associations or short-term prediction performance and provide limited assessment of indirect or mediating pathways among climate, population, vector, and disease variables. In addition, most previous models do not explicitly account for spatial heterogeneity across administrative levels or test whether structural relationships differ between ecological settings, such as inland and coastal regions. Conventional regression and forecasting models may also have limited ability to simultaneously assess latent constructs, measurement validity, and multigroup structural differences within integrated surveillance systems. Despite these advances, critical knowledge gaps remain regarding how key determinants vary across spatial and administrative levels. National-level responses often adopt uniform strategies, yet local dynamics can differ significantly between districts and governorates. To address this, the present study applies Partial Least Squares Structural Equation Modeling (PLS-SEM) with Multigroup Analysis (MGA) to explore and compare the structural pathways linking Weather, population, and vector indicators to VBD incidence across three governorates, such as Muscat, North Al Batinah, and Ad Dakhiliyah, and their corresponding districts, including Seeb, Sohar, and Bahla [14,15]. Recent regional studies have demonstrated the strength of PLS-SEM in modeling complex, multi-dimensional public health outcomes using latent constructs and indirect pathways, even with moderate sample sizes [16,17]. Building on this framework, we apply a similar structural approach at both the governorate and district levels to identify spatially nuanced drivers of dengue outbreaks [18].

The present study extends previous work by integrating PLS-SEM with MGA and Measurement Invariance of Composite Models (MICOM) to compare dengue transmission pathways across multiple ecological and demographic settings in Oman. Unlike previous single-setting or prediction-focused studies, this framework simultaneously evaluates direct, indirect, and mediating relationships among climate, population, vector, and dengue indicators while allowing formal comparison between governorate- and district-level transmission structures. The proposed approach also offers methodological advantages for handling non-normal surveillance data, addressing multicollinearity, handling moderate sample sizes, and latent-variable modeling within complex public health systems.

This work is grounded in Rothman’s “epidemic pie” model and previous literature [19] and represents both a methodological and spatial extension of the previously validated integrated PLS-SEM framework developed in Seeb, Oman [12]. While the earlier study focused on a single district in an exploratory analysis, the current study expands the framework to multiple governorates and districts, incorporates Multigroup Analysis (MGA) and MICOM-based measurement invariance testing, and evaluates geographical heterogeneity in dengue transmission pathways using longitudinal surveillance data collected between 2020 and 2024. Our primary aim is to investigate how direct and indirect effects vary across different spatial scales in Oman. The secondary aim is to explore geographically distinct transmission patterns and structural relationships of dengue outbreaks and inform more targeted public health interventions. This study provides one of the first multigroup structural comparisons of dengue transmission dynamics across governorate and district levels in Oman using PLS-SEM. The study identifies geographically distinct direct and indirect transmission pathways across inland and coastal settings, highlights the operational value of vector indicators for localized surveillance and early warning, and provides evidence to support geographically tailored vector control and outbreak response strategies in Oman. Therefore, the objective of this study is to evaluate and compare the structural relationships among climate factors, population demographics, mosquito vector indicators, and dengue incidence using PLS-SEM-MGA across governorate and district levels in Oman [14,15].

**Figure 1:**
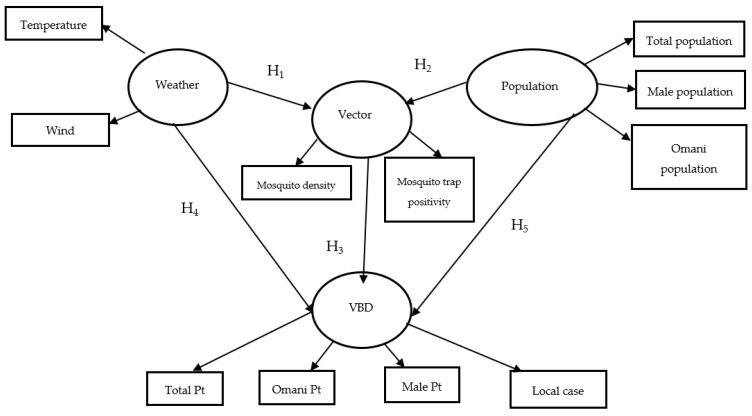
The Conceptual Model. Ellipse: Latent variables; Rectangle: Manifest variables; VBD, Vector-borne disease. Five hypotheses in the conceptual model (H_1_: Weather → Vector; H_2_: Population → Vector; H_3_: Vector → VBD; H_4_: Weather → VBD via Vector: H_5_: Population → VBD via Vector).

The conceptual model was developed to assess the direct and indirect relationships among climate, population, vector dynamics, and dengue transmission (Figure 1). Its design was informed by findings from previous modeling efforts in Seeb [12] and Rothman’s multifactorial causation model [20]. The model posits that climatic and population factors affect dengue incidence indirectly by influencing the occurrence of the mosquito vector [2,21], with five hypotheses tested to evaluate these relationships as listed below:

**H_1_**: *It was hypothesized that weather conditions, particularly temperature and wind, would positively influence mosquito survival and activity (H_1_: Weather → Vector).*

**H_2_**: *Population characteristics such as urban density and the presence of migrant labor were expected to affect mosquito breeding and habitat suitability (H_2_: Population → Vector) [8].*

**H_3_**: *Increased mosquito trap positivity and mosquito density are positively associated with higher dengue transmission rates (H_3_: Vector → VBD).*

**H_4_**: *Weather affects dengue incidence indirectly via its influence on vectors (H_4_: Weather → VBD via Vector).*

**H_5_**: *Population factors impact dengue transmission through their effect on mosquito abundance and human exposure risk (H_5_: Population → VBD via Vector).*

All the above hypotheses reflect the contextual realities of dengue outbreaks in Oman, particularly since 2018, when locally transmitted cases began to emerge in diverse ecological regions [9,16]. Given the limited number of governorates and districts included, the present analysis should be interpreted as exploratory and hypothesis-generating rather than nationally representative.

## 2. Materials and Methods

### 2.1. Selected Governorates and Districts in Oman

The selected governorates represent diverse geographic, demographic, and ecological settings within Oman, which is important for understanding variations in vector-borne disease transmission patterns [12]. According to the 2024 National Center for Statistics and Information (NCSI) report, Muscat is the most densely populated and highly urbanized coastal area, with approximately 1.47 million residents and a predominantly non-Omani population (58%). North Al Batinah Governorate has about 926,000 residents and moderate urbanization, linked to its industrial and port activities. Ad Dakhiliyah Governorate is an inland rural region with dispersed settlements, the smallest population (about 559,000), and the lowest population density (15 people/km^2^) among the three governorates

To enable localized comparison within each governorate, one district was selected based on ecological representativeness, population size, and dengue case burden. While NCSI does not publicly release official district-level density data, local estimates provide important insight into demographic characteristics. Seeb (Muscat Governorate) is the most populous district (368,096), with a majority non-Omani population (62.5%) and a very high inferred population density. It is a key coastal urban center with dense residential and commercial activity. Sohar (North Batinah Governorate) has a population of 203,265, with a predominantly Omani population (65.2%) and a moderate urban–industrial density. It serves as a vital industrial port city. Bahla (Ad Dakhiliyah Governorate) is the least populated (105,313), has the highest percentage of Omanis (76.5%), and features a low rural–periurban density. It remains a traditional inland town with limited urban expansion. These demographic and ecological differences are relevant for interpreting the observed variations in the PLS-MGA results, particularly along the Population → Vector and Population → VBD pathways. For example, Seeb’s high expatriate population and dense housing may increase vector exposure, while Bahla’s rural characteristics may affect vector ecology differently.

The selected governorates and districts were purposively chosen to reflect diverse ecological, demographic, and epidemiological settings across Oman, including highly urbanized coastal areas, industrial port environments, and inland rural and peri-urban regions. These contrasting settings provided an opportunity to explore how climate, population structure, and vector dynamics may interact differently across varying transmission environments. In addition, these locations had the most complete and consistent longitudinal epidemiological and vector surveillance datasets available during the study period, allowing more reliable multigroup structural comparisons. Accordingly, the comparative framework was primarily explanatory, designed to investigate potential spatial heterogeneity in dengue transmission pathways across different administrative and ecological contexts, and to generate evidence to guide future nationwide analyses and model expansion.

### 2.2. Data Sources and Variables

Two related but distinct analytical datasets were developed for this study. The first dataset consisted of weekly aggregated governorate-level data for the Muscat, North Al Batinah, and Ad Dakhiliyah Governorates. The second dataset consisted of district-level weekly data for the Seeb, Sohar, and Bahla districts, selected as representative ecological and epidemiological settings within their respective governorates. Separate PLS-SEM and MGA analyses were subsequently performed for the governorate-level and district-level datasets to compare structural relationships across different administrative scales.

Data for this study were compiled from three authoritative national sources: the Ministry of Health (MOH), the Directorate General of Meteorology under the Civil Aviation Authority, and the NCSI. The dataset comprised weekly aggregated records collected between January 2020 and December 2024, as available, and encompassed variables related to dengue cases, mosquito surveillance, climate conditions, and population demographics. Dengue case data were obtained from MOH’s electronic surveillance systems and included weekly counts of total confirmed dengue cases, locally transmitted (autochthonous) cases, male patients, and Omani patients. The analysis did not exclude females or non-Omani patients, as total population and total dengue case indicators were also incorporated within the latent constructs. The inclusion of male and Omani population/patient indicators was primarily intended to capture gender and nationality dimensions within the model while reducing multicollinearity among highly correlated demographic variables. These indicators were therefore retained for statistical and epidemiological representation purposes rather than to limit the analysis to specific population groups. Vector surveillance indicators were collected through routine monitoring using gravid mosquito traps, including the trap positivity rate (the percentage of traps that were positive) and mosquito density (the average number of mosquitoes per trap). Population data, sourced from NCSI, included estimates of total population, Omani nationals, and male residents at the district level. Climatic data were provided by the Directorate General of Meteorology and included weekly measurements of temperature (°C), wind speed (km/h), rainfall (mm), and relative humidity (%). While all four climatic variables were initially incorporated into preliminary models, relative humidity was excluded from the final model due to low factor loadings observed during measurement model assessment [12,22]. All variables were compiled at the district level and recorded weekly. The data underwent preprocessing steps, including time-series imputation for missing values, variable standardization, and aggregation by epidemiological week to ensure consistency and compatibility for subsequent statistical analyses [23].

### 2.3. Measurement Model and Constructs

The measurement model specified four reflective latent constructs capturing the major domains of dengue transmission: vector presence, climatic drivers, population exposure, and disease burden. These constructs were defined based on prior literature, national surveillance experience, and an exploratory factor analysis conducted with SmartPLS 4.0 [24]. Each latent construct was modeled reflectively. A reflective specification was selected because the latent constructs were assumed to drive variation in the observed surveillance indicators, consistent with the theoretical framework of dengue transmission and previous PLS-SEM applications in public health research [25]. The merit of the proposed measurement model lies in its ability to integrate multiple surveillance dimensions (climate, demographic, vector, and disease indicators) into latent constructs representing real-world transmission systems. This approach reduces measurement error, captures indirect ecological relationships, and enables the simultaneous assessment of complex pathways that cannot be adequately evaluated with conventional regression models. The constructs and their associated indicators are shown in Table 1. These indicators were derived from weekly surveillance data collected between 2020 and 2024. Missing vector data were imputed using Random Forest models with high predictive accuracy (coefficient of determination (R^2^) > 0.89), and the imputed values were flagged for transparency. Linear interpolation was applied to address short-term gaps in climate data. The model’s reliability and validity were assessed using standard criteria, including indicator loadings above 0.70, an average variance extracted (AVE) greater than 0.50, and composite reliability above 0.70. Discriminant validity was confirmed through both the Fornell–Larcker criterion and Heterotrait–Monotrait (HTMT) ratios below 0.85 [22,26]. The proposed model consists of four functional components. The first component is the environmental driver component, represented by weekly weather indicators, particularly temperature and wind speed. The second component is the population exposure component, represented by the total population, male population, and Omani population indicators. The third component is the vector surveillance component, represented by mosquito trap positivity and mosquito density, which functions as the mediating ecological pathway linking environmental and demographic conditions to disease occurrence. The fourth component is the disease outcome component, represented by total confirmed dengue cases, local cases, male cases, and Omani cases. Within this architecture, weather and population indicators are hypothesized to indirectly influence dengue transmission by affecting vector abundance. In contrast, the vector construct is hypothesized to directly affect VBD incidence. The same model architecture was consistently applied across governorate- and district-level datasets to enable a valid multigroup comparison after assessment of measurement invariance.

### 2.4. Statistical Analysis

Data analysis was conducted using Partial Least Squares Structural Equation Modeling (PLS-SEM) in SmartPLS v4.0 [24]. This method was selected for its ability to handle complex theoretical models involving multiple constructs, moderate sample sizes, and non-normally distributed data. PLS-SEM also enables the simultaneous estimation of both measurement and structural models, which is particularly suitable for exploratory and theory-driven research in public health contexts [27]. Compared with conventional regression or forecasting approaches, PLS-SEM additionally allows simultaneous evaluation of direct, indirect, and mediating relationships among multiple latent constructs while accounting for measurement reliability and structural heterogeneity across groups. The integration of MGA and MICOM further enabled formal comparison of transmission pathways between different ecological and administrative settings. The assessment of the measurement model included evaluation of internal consistency reliability through Cronbach’s Alpha (CA) and composite reliability (CR), with acceptable values set at greater than 0.70 [28]. Convergent validity was established through an average variance extracted (AVE) of greater than 0.50 and outer loadings above 0.70 [29]. Discriminant validity was verified using the HTMT ratios, which were below 0.85 [26]. For the structural model, path coefficients (β) and their significance were estimated via bootstrapping with 5000 subsamples [30]. Additionally, the explanatory power of endogenous constructs was assessed using coefficient of determination (R^2^) values, and effect sizes (f^2^) were reported to indicate the relative contribution of predictors. Direct, indirect, and total effects were computed to evaluate mediation effects and validate the theoretical framework. Model fit was evaluated by comparing saturated and estimated models using the Standardized Root Mean Square Residual (SRMR), Squared Euclidean Distance (d_ULS), Geodesic Distance (d_G), and Normed Fit Index (NFI). The recommended cut-off values for SRMR and NFI were <0.08 and >0.90, respectively. The lower values of d_ULS and d_G are preferable for the chosen model.

To examine whether the model behaved differently across administrative and ecological contexts, an MGA was conducted using the permutation method in SmartPLS, with 1000 permutations and a significance threshold of 1.67% (=5%/3) [14,31]. The analysis was performed across three governorates (Muscat, North Batinah, and Ad Dakhiliyah) in Oman and three districts, namely Seeb in Muscat, Sohar in North Batinah, and Bahla in Ad Dakhiliyah, which were selected based on their ecological diversity and dengue burden. Before proceeding with MGA, the MICOM procedure was applied to ensure that comparisons across groups were valid [25,31]. MICOM involves three sequential steps. First, configural invariance was established by confirming that the model structure, data treatment, and algorithm settings were identical across groups. Second, compositional invariance was tested using a permutation procedure, with a target correlation of at least 0.95 indicating equivalence [31]. Third, the equality of mean and variance was evaluated to determine whether full or partial measurement invariance had been achieved. MGA results were interpreted only when measurement invariance was established. Significant differences in path coefficients across groups were used to identify contextual variations in the strength and direction of relationships among the climate, population, vector, and disease constructs.

The MGA findings were interpreted cautiously as exploratory comparisons intended to identify potential structural heterogeneity across different ecological and administrative settings rather than establish definitive causal regional differences. The comparative framework was designed to examine how transmission pathways may vary between contrasting governorate and district contexts and to support hypothesis generation for future expanded analyses.

## 3. Results

### 3.1. Descriptive Trends of Dengue Surveillance Data

**Figure 2:**
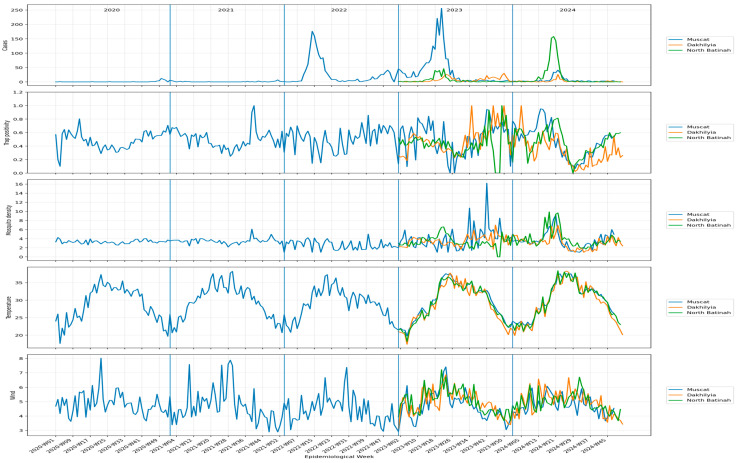
Weekly confirmed dengue cases and associated vector and weather indicators by governorate, 2020–2024.

**Figure 3:**
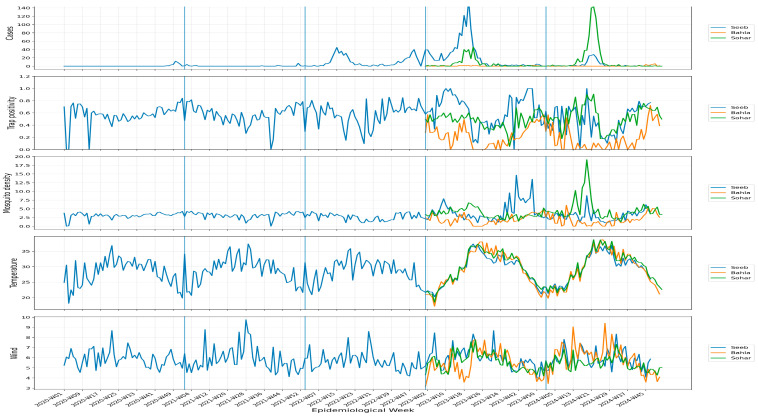
Weekly confirmed dengue cases and associated vector and weather indicators by Districts, 2020–2024.

Figure 2 and Figure 3 show the weekly distribution of confirmed dengue cases across the selected governorates and districts from 2020 to 2024. At the governorate level, Muscat showed the largest dengue peaks, particularly during 2022 and 2023, while North Batinah showed a marked increase in 2024. Ad Dakhiliyah showed lower overall case counts with smaller peaks. At the district level, Seeb recorded the highest dengue activity, especially in 2023, whereas Sohar had a large peak in 2024. Bahla reported comparatively low weekly case counts throughout the study period. These descriptive trends indicate spatial and temporal heterogeneity in dengue transmission across the selected areas and provide context for the subsequent PLS-SEM and multigroup analyses.

### 3.2. Measurement Model Assessment

The measurement model is evaluated across governorates and districts using R^2^, f^2^, CR, CA, AVE, and outer loadings. Table 2 summarizes key measurement quality statistics for governorates and districts. All constructs demonstrated acceptable reliability and validity, with CRs > 0.87 and AVEs > 0.61 across all groups. Outer loadings ranged from 0.857 to 1.000, indicating high indicator reliability. At the governorate level, R^2^ values for the Vector construct ranged from 0.125 (Muscat) to 0.390 (North Batinah), and for VBD from 0.002 (Muscat) to 0.190 (Ad Dakhiliyah), indicating substantial variability in model explanatory power. At the district level, R^2^ values indicated stronger explanatory power for VBD in Bahla (0.190) and Sohar (0.160), whereas in Seeb, it was very low (R^2^ = 0.006). To summarize, all key differences in R^2^, f^2^, and measurement-quality statistics across governorates and districts are presented. These results confirm acceptable construct reliability and convergent validity in all groups, while also highlighting statistically significant differences in population and VBD measurement quality, particularly between Muscat and Ad Dakhiliyah, and between Seeb and Bahla. This finding supports the need to interpret multigroup structural comparisons cautiously, especially when the underlying measurement strength varies across regions.

### 3.3. The Final Model Evaluation Indices

To further assess construct stability across regions, pairwise comparisons were performed. The structural model was assessed using standardized path coefficients (β) and evaluated using permutation testing (5000 resamples) [23,30].

#### 3.3.1. Governorate-Level Analysis

**Figure 4:**
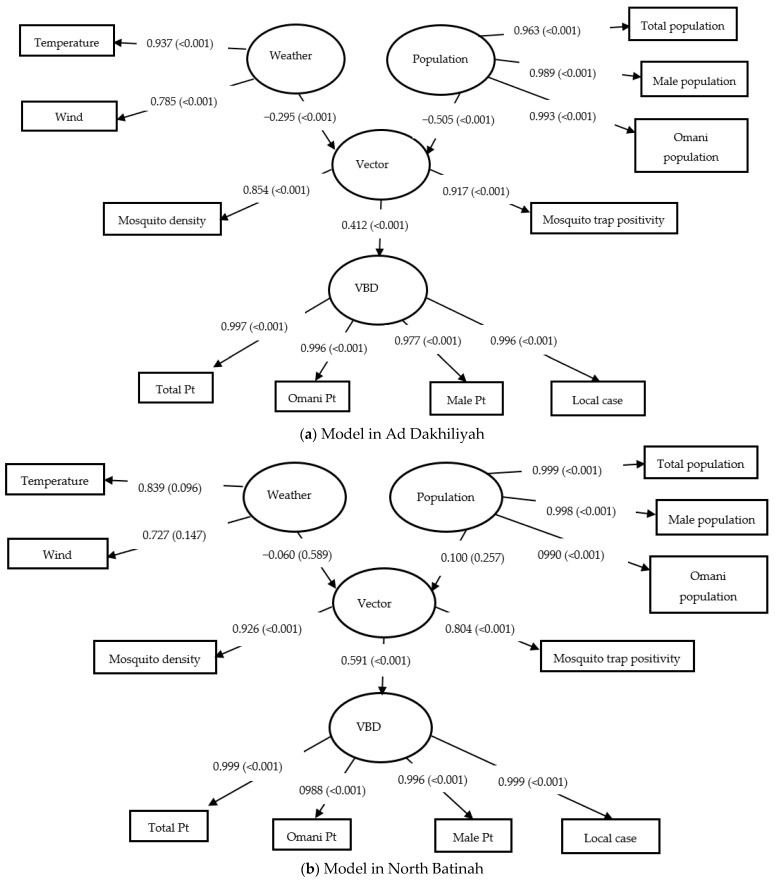

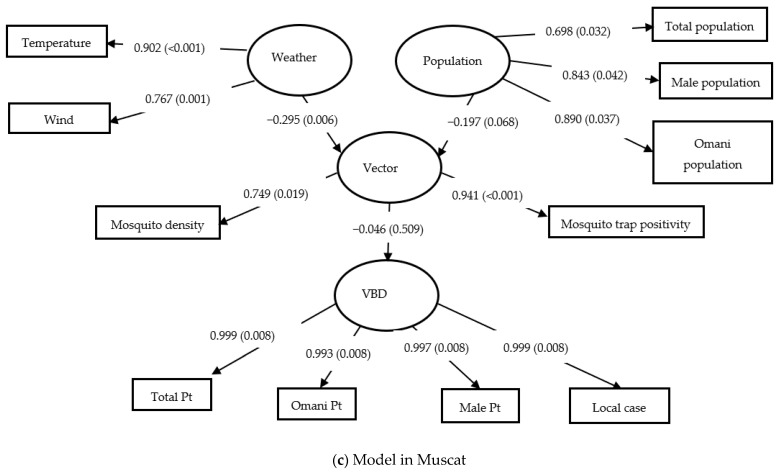
Structural Model in Three Governorates in Oman.

Table 3 presents the structural path coefficients for Muscat, North Batinah, and Ad Dakhiliyah, and all three models are displayed graphically in Figure 4 (data available in Supplementary Materials). The path from Vector → VBD was strongest in Ad Dakhiliyah (β = 0.436) and significantly different from Muscat (β = −0.021) with a β difference of 0.457 (*p*< 0.001). The indirect effects from both Population on VBD (β diff = −0.267, *p* < 0.001) and Weather on VBD (β diff = −0.135, *p* < 0.001) were significantly higher in Dakhiliya compared to Muscat and North Batinah, respectively. The Population → Vector path differed significantly between Dakhiliya and North Batinah (β diff = −0.605, *p* < 0.001), while no significant difference was observed in Weather → Vector (*p* > 0.0167) across governorates.

#### 3.3.2. District-Level Analysis

**Figure 5:**
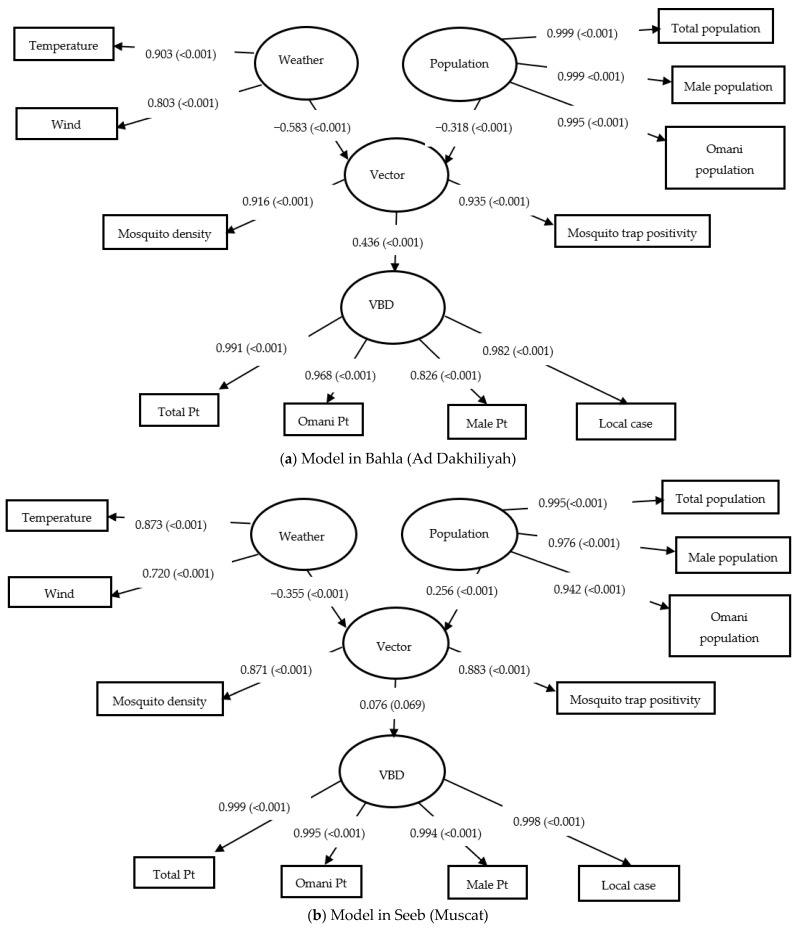

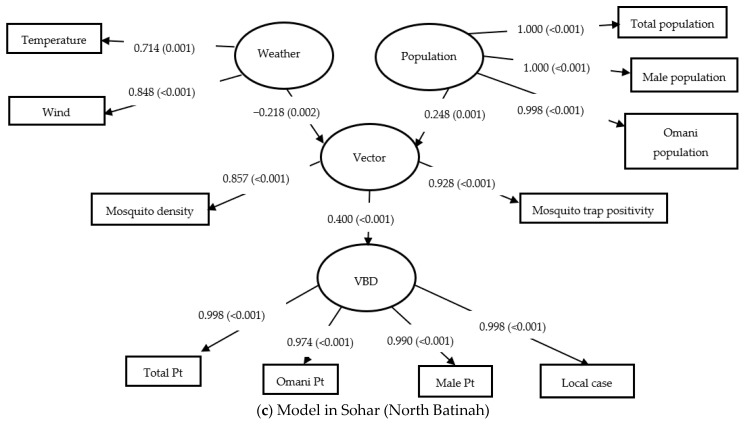
Structural Model in Three Districts.

At the district level, the structural models were compared for Seeb (Muscat), Sohar (North Batinah), and Bahla (Ad Dakhiliyah), and their results are displayed graphically in Figure 5 (data available in Supplementary Materials). Table 3 summarizes the structural path coefficients and group differences. The Vector → VBD path was significantly stronger in Bahla (β = 0.436) than in Seeb (β = 0.076), with a β difference of 0.359 (*p* <0.001). Similarly, Weather → Vector (β diff = −0.365, *p* = 0.001) and Population → Vector (β diff = −0.566, *p* < 0.001) paths were significantly stronger in Bahla than in Sohar. The indirect effects from both Population and Weather on VBD also showed substantial differences, particularly between Bahla and Sohar (Population → VBD: β diff = −0.238, *p* < 0.001; Weather → VBD: β diff = −0.167, *p* < 0.001). These findings highlight stronger vector-mediated effects in Bahla and Sohar, particularly in path strength and indirect effects, even though the overall R^2^ values between Bahla and Sohar were not significantly different. In contrast, Seeb showed limited predictive capacity across all structural paths.

### 3.4. Multigroup Analysis and MICOM Results

An MGA was conducted using permutation testing (5000 resamples) to examine whether path coefficients, means, and variances significantly differ across governorates and districts. Prior to MGA, the MICOM procedure was applied to test for measurement invariance in three steps: configural invariance, compositional invariance (Step 2), and equality of means (Step 3a) and variances (Step 3b) [14,23,31]. Table 4 summarizes the MICOM assessment across the districts and governorates. All group comparisons showed configural invariance, as the model structure and indicators were identical across groups. Compositional invariance (Step 2) was partially achieved in most cases. However, Step 3 revealed significant differences in means and variances for several constructs, especially for Population and VBD, across almost all comparisons. These results confirm partial measurement invariance, permitting valid multigroup comparisons with caution.

### 3.5. Discriminant Validity and Model Fit Assessment

Discriminant validity was evaluated using the HTMT criterion. In the saturated model, the HTMT values of all 6 pairs of constructs were below the conservative threshold of 0.85 (Population ↔ VBD: 0.199, Vector ↔ VBD: 0.154, Vector ↔ Population: 0.125, Weather ↔ VBD: 0.219, Weather ↔ Population: 0.145, Weather ↔ Vector: 0.555), confirming discriminant validity between constructs. Model fit was evaluated by comparing the estimated and saturated models using Standardized Root Mean Square Residual (SRMR), Squared Euclidean Distance (d_ULS), Geodesic Distance (d_G), and Normed Fit Index (NFI) indices. The SRMR values for the saturated and estimated models were 0.058 and 0.103, respectively, both below the recommended cut-off of 0.08, indicating acceptable model fit. The d_ULS value of the saturated (0.222) model is lower than that of the estimated (0.706) model. The d_G value of the saturated (0.943) model is lower than that of the estimated (0.956) model. The NFI value for the saturated model (0.842) is higher than that for the estimated model (0.838), and the difference is marginal but exceeds the acceptable level (>0.90). These results support the overall adequacy of the model, particularly in the saturated configuration, despite some estimate inflation due to indicator redundancy within the VBD construct.

## 4. Discussion

This study employed PLS-SEM with MGA to investigate the impact of climate, population, and vector indicators on dengue incidence across various administrative levels in Oman. It represents one of the few national efforts to directly compare structural disease drivers at both the governorate and district levels using multigroup path modeling [14,15]. The findings of this study should be interpreted within the context of an exploratory multigroup structural framework designed to examine potential spatial heterogeneity in dengue transmission pathways across contrasting ecological and administrative settings in Oman. The comparisons among governorates and districts were intended to identify possible differences in structural relationships rather than establish definitive causal regional effects. Accordingly, the observed associations should be considered hypothesis-generating and may support future nationwide analyses incorporating additional geographic, demographic, behavioral, and mobility-related variables.

### 4.1. Interpretation of Key Findings

The results confirmed that mosquito abundance mediates the influence of climate and demographic variables on dengue outbreaks. However, the significance and magnitude of these effects varied considerably by region. At the governorate level, Dakhiliya exhibited the strongest vector-mediated disease pathway (Vector → VBD: β = 0.436), indicating the ecological sensitivity of this inland governorate. Muscat, despite being the highest-burdened area in national surveillance data (3795 dengue cases between 2018 and 2025), had weak structural associations (R^2^ VBD = 0.002), potentially due to high population mobility, past exposure, or partial herd immunity that reduces the variation captured by ecological indicators [3,4]. The Seeb district, the origin of local transmission in Oman, showed similarly low model fit (R^2^ VBD = 0.006), despite its high population density and historically elevated case counts. These findings are consistent with prior PLS-SEM analysis conducted in Seeb [12], which found that population factors (β = 0.231) had a greater total effect on dengue transmission than vector indicators (β = 0.116), and climatic effects were largely indirect. This finding may reflect stronger social mobilization, public health preparedness, and routine vector control efforts in areas previously affected by outbreaks, as compared to newly affected regions. In contrast, Bahla and Dakhiliya showed significant structural sensitivity to climate and vector dynamics. Bahla had the strongest Weather → Vector relationship (β = −0.583), reinforcing the idea that rural highland districts remain structurally vulnerable to climate-induced vector proliferation and should be prioritized for early warning systems. Biologically, higher R^2^ values in Bahla and Ad Dakhiliyah suggest that ecological and vector-related factors more strongly explain dengue transmission in these inland settings. In contrast, the very low R^2^ values observed in Muscat and Seeb suggest that additional unmeasured determinants, such as mobility, human behavior, and urban complexity, may contribute substantially to transmission dynamics. Similarly, larger f^2^ values for the Vector → VBD pathway support the biological importance of mosquito abundance and trap positivity as operational indicators of dengue transmission risk.

### 4.2. Reinforcing Evidence from National Modeling Studies

These results align with the Bayesian prediction model study by our research team, which showed that mosquito trap positivity was the most consistent predictor of dengue across Oman. At the same time, temperature and wind had significant lagged effects [13]. Rainfall and humidity were also excluded in that study due to a lack of significance, confirming the findings here. Their best-performing model, a Negative Binomial regression with lagged variables, achieved an Area Under the Curve (AUC) of 0.881, confirming that entomological and delayed climatic factors are central to accurate prediction of outbreaks [18]. That study also emphasized spatial heterogeneity and the importance of district-level stratification, validating the approach used here to separate Sohar, Bahla, and Seeb for structural comparisons.

### 4.3. Contextualizing Structural Variation Across Regions

Several factors may explain the observed differences in model strength and path significance. Muscat’s high population density (355/km^2^), expatriate population (58%), and history of multiple outbreaks suggest evolving immunity, adaptation, or saturation effects that reduce measurable structural influence. Seeb’s urban infrastructure, expatriate dominance, and high mobility rates (62.5% non-Omani residents) likely contribute to low explanatory power in traditional ecological models and necessitate the inclusion of mobility and behavioral variables in future studies [13]. Dakhiliya and Bahla’s inland geography, low population density, and climate sensitivity make them more responsive to environmental drivers, supporting the need for climate-based forecasting tools in rural districts. These structural patterns are supported by previous studies [12], which highlighted the role of socioeconomic disparities, health literacy, and vector control engagement in shaping dengue burden [5].

### 4.4. Strengths and Limitations

This study used PLS-SEM to model complex latent relationships and indirect effects, a methodology suitable for moderate sample sizes and imperfect data. The MGA component provided valuable statistical comparison across districts and governorates, identifying meaningful spatial variability [14,32]. All constructs (CR > 0.87, AVE > 0.61, Outer loadings mostly > 0.85, and HTMT ratios < 0.85) showed strong measurement validity. MICOM supported partial measurement invariance, enabling comparative interpretation. The approach is consistent with best practices in environmental health modeling and complements previous hierarchical models in Oman [13].

This study has several limitations that may affect the results. Rainfall and humidity were excluded due to weak factor loadings; however, delayed effects may still be important. Mobility data, a key dengue risk factor in urbanized regions like Muscat and Seeb, were not included [8]. Missing data required imputation, which may introduce bias [23]. Only two entomological indicators (density and trap positivity) were included; future models should also include larval indices, adult captures, and resistance profiles. Behavioral and socioeconomic factors (e.g., health-seeking behavior, housing quality, awareness campaigns) were not modeled, despite their likely importance in areas like Seeb [5,12]. In addition, the comparative framework included a selected group of governorates and districts representing contrasting ecological and demographic settings in Oman. Although this approach enabled exploratory multigroup structural comparisons, future studies incorporating additional regions and higher-resolution spatial data may provide a broader understanding of regional heterogeneity in dengue transmission dynamics.

## 5. Conclusions

The present study applied PLS-SEM with Multigroup Analysis (MGA) to explore structural relationships among climate, population, vector, and dengue indicators across selected governorates and districts in Oman. The findings demonstrated substantial spatial variability in the strength and direction of dengue transmission pathways across contrasting ecological and administrative settings. Although Muscat and North Batinah showed lower explanatory power within the current structural framework, they remain important surveillance and early warning settings due to high population density, urban complexity, and expatriate population dynamics [3,4]. In contrast, Ad Dakhiliyah and Bahla demonstrated relatively stronger climate- and vector-related structural associations, suggesting potential value for enhanced climate-informed forecasting and entomological surveillance in inland regions [7].

The findings also suggest that vector indicators, particularly mosquito trap positivity and mosquito density, may serve as important operational indicators for district-level dengue surveillance and early warning systems [13]. In addition, the results support the potential value of geographically tailored intervention approaches that incorporate seasonal risk assessment, community-specific health communication, and the integration of mobility, demographic, and socio-behavioral information into public health surveillance systems [10,11].

The present framework should be interpreted as an exploratory, hypothesis-generating multigroup analysis to investigate potential spatial heterogeneity in dengue transmission pathways across different ecological contexts in Oman. Future studies should expand the model by incorporating additional governorates and districts, mobility and socioeconomic variables, intervention-related indicators, and higher-resolution spatial and temporal datasets. Integration of PLS-SEM with Bayesian forecasting and machine-learning approaches may further strengthen predictive performance and support the development of operational early warning systems for vector-borne diseases in Oman.

## Figures and Tables

**Table 1:** Latent Constructs and Their Indicators.

Latent Construct	Indicators
Vector	Mosquito trap positivity (%), Mosquito density (average per trap)
Weather	Temperature (°C), Wind speed (km/h)
Population	Total population, Male population, Omani population
VBD	Total confirmed dengue cases, Local (autochthonous) cases, Male dengue cases, Omani dengue cases

**Table 2:** Measurement Model Comparison within the Governorate and District Level.

Comparison	R^2^ VBD (Diff, *p*-Value)	f^2^ Vector → VBD (Diff, *p*-Value)	f^2^ Population → Vector (Diff, *p*-Value)	AVE Population (Diff, *p*-Value)	CA Population (Diff, *p*-Value)
Governorates					
Ad Dakhiliyah vs. Muscat	0.167, *p* < 0.001	0.202, *p* < 0.001	0.318, *p* = 0.005	0.301, *p* = 0.121	0.204, *p* < 0.001
Ad Dakhiliyah vs. North Batinah	−0.180, *p* = 0.291	−0.333, *p* = 0.305	0.352, *p* < 0.001	−0.027, *p* = 0.001	−0.014, *p* < 0.001
Muscat vs. North Batinah	0.347, *p* < 0.001	0.534, *p* < 0.001	−0.034, *p* = 0.763	0.328, *p* = 0.191	0.218, *p* < 0.001
Districts (Governorates)					
Seeb (Muscat) vs. Sohar(North Batinah)	0.154, *p* = 0.007	0.185, *p* = 0.008	−0.103, *p* = 0.264	−0.013, *p* = 0.371	−0.004, *p* = 0.377
Seeb (Muscat) vs. Bahla (Ad Dakhiliyah)	0.184, *p* < 0.001	0.228, *p* < 0.001	0.390, *p* < 0.001	−0.102, *p* = 0.003	−0.039, *p* < 0.001
Bahla (Ad Dakhiliyah) vs. Sohar (North Batinah)	0.030, *p* = 0.817	0.044, *p* = 0.820	0.494, *p* < 0.001	−0.089, *p* < 0.001	−0.035, *p* < 0.001

VBD, Vector-borne disease; Diff, difference between the two latent constructs; R^2^, Coefficient of Determination; f^2^, Effect sizes; AVE, Average Variance Extracted; CA, Cronbach’s Alpha.

**Table 3:** Structural Path Coefficients * among Governorates and Districts in Oman.

Governates	Muscat	North Batinah	Ad Dakhiliyah	β Difference (*p*-Value)
Population → Vector	−0.326	0.248	−0.318	Ad Dakhiliyah vs. North Batinah: −0.605 (*p* < 0.001)
Weather → Vector	−0.401	−0.218	−0.583	Not significant (all *p* > 0.0167)
Vector → VBD	−0.021	−0.057	0.436	Ad Dakhiliyah vs. Muscat: 0.457 (*p* < 0.001)
Population → VBD (indirect)	−0.127	0.099	−0.138	Ad Dakhiliyah vs. North Batinah: −0.267 (*p* < 0.001)
Weather → VBD (indirect)	−0.201	−0.087	−0.254	Ad Dakhiliyah vs. Muscat: −0.135 (*p* < 0.001)
Districts	Seeb	Sohar	Bahla	
Population → Vector	0.256	0.248	−0.318	Bahla vs. Sohar: −0.566 (*p* < 0.001)
Weather → Vector	−0.401	−0.218	−0.583	Bahla vs. Sohar: −0.365 (*p* = 0.001)
Vector → VBD	0.076	0.400	0.436	Seeb vs. Bahla: 0.359 (*p* < 0.001)
Population → VBD (indirect)	−0.127	0.099	−0.138	Bahla vs. Sohar: −0.238 (*p* < 0.001)
Weather → VBD (indirect)	−0.201	−0.087	−0.254	Bahla vs. Seeb: −0.227 (*p* < 0.001)

* Not all pair comparisons were shown because they are not significant at a 1.67% alpha level; VBD, Vector-borne disease.

**Table 4:** Measurement Invariance of Composite Models (MICOM) Assessment on the Districts and Governorates.

Comparison	VBD (Step 2/3a/3b)	Population (Step 2/3a/3b)	Vector (Step 2/3a/3b)	Weather (Step 2/3a/3b)	Invariance Status
Ad Dakhiliyah vs. Muscat	0.999/<0.001/<0.001	0.820/<0.001/<0.001	0.891/0.562/0.897	0.835/0.849/0.116	Partial
Ad Dakhiliyah vs. North Batinah	0.277/0.121/0.015	0.781/<0.001/<0.001	0.100/0.437/0.632	0.821/0.238/0.004	Partial
Muscat vs. North Batinah	0.623/0.237/0.448	0.823/<0.001/<0.001	0.923/0.192/0.891	0.893/0.199/0.293	Partial
Seeb vs. Sohar	0.615/0.404/0.358	0.961/<0.001/<0.001	0.710/0.212/0.472	0.279/0.477/0.742	Partial ^
Seeb vs. Bahla	0.372/<0.001/<0.001	0.758/<0.001/<0.001	0.763/<0.001/<0.001	0.947/0.429/<0.001	Partial
Bahla vs. Sohar	0.184/<0.001/<0.001	0.063/<0.001/<0.001	0.611/<0.001/0.004	0.298/0.864/<0.001	Partial

Step 2, *p*-value on test for compositional invariance; Step 3a, *p*-value on test for equality of means; Step 3b, *p*-value on test for equality of variances; ^, no full 3-step pass; VBD, Vector-borne disease.

## Data Availability

The data presented in this study are available in this article (and Supplementary Material).

## Supplementary Materials

The following supporting information can be downloaded at: https://www.mdpi.com/article/10.3390/diseases14060196/s1, Table S1: Governorate; Table S2: District.
